# Comparison of Surface Modification Methods for Improving the Compatibility of Recycled Plastic Film-Based Aggregates

**DOI:** 10.3390/polym13223956

**Published:** 2021-11-16

**Authors:** Jea Uk Lee, Jin-Yong Hong

**Affiliations:** 1 Department of Advanced Materials Engineering for Information & Electronics, Kyung Hee University, Yongin-si 17104, Gyeonggi-do, Korea; leeju@khu.ac.kr; 2 Center for C1 Gas & Carbon Convergent Research, Korea Research Institute of Chemical Technology (KRICT), 141 Gajeong-ro, Yuseong-gu, Daejeon 34114, Korea

**Keywords:** plastic film waste, recycling, concrete aggregates, surface modification, hydrophilicity

## Abstract

The surface modification of recycled plastic film-based aggregates was investigated to improve the compatibility between the aggregates and a cement paste. Surface modification was performed using ultraviolet–ozone treatment (UV-O_3_), a silane coupling agent, O_2_ atmospheric pressure plasma, and acrylic binder coating methods. The surface properties of the modified aggregates were analyzed using a contact angle measuring instrument. The results revealed that for all surface modification methods, the contact angle decreased with an increase in the treatment time. According to the comparative evaluation results of the changes in the surface characteristics of the aggregates through various surface modification methods, the contact angle reduction rates were 58.9%, 51.4%, 25.5%, and 24.5% for the O_2_ atmospheric pressure plasma, the acrylic binder coating, the silane coupling agent, and the UV-O_3_ method, respectively. After 48 h, the contact angle had increased by 110.9%, 29.9%, 16.4%, and 5.9% for the O_2_ atmospheric pressure plasma, UV-O_3_, the silane coupling agent, and the acrylic binder coating, respectively. Namely, the surface modification using the acrylic binder coating method was found to be the most effective method in terms of the wettability increase effect and the long-term storage stability.

## 1. Introduction

Since the invention of Bakelite, the first synthetic plastic, in 1907, plastics have emerged as alternatives to ceramics, wood, glass, and metal and have been developed in various forms. Plastic is a material developed by supplementing the shortcomings of existing materials and maximizing their advantages and has significantly contributed toward a rich human life and the industrial development of modern times owing its excellent functionality and low price. However, although its excellent chemical stability is its most significant advantage, it does not rust or decay, leading to a new concern with regard to global ecosystem pollution [1].

Currently, waste plastics are treated using methods such as landfilling, incineration, and recycling. In the case of treatment through landfilling, there are concerns regarding secondary environmental pollution, such as the significantly long decomposition time of plastic, the leaching of environmental hormones due to landfilling, and the generation of microplastics during the decomposition of plastic. The incineration treatment method also has a fatal impact on the ecosystem due to the emission of dioxin, which is a carcinogen, volatile organic compounds (VOCs), which are air pollutants, and carbon dioxide (CO_2_), which is the main culprit in greenhouse gases during incineration. Waste plastics that cannot be recycled and are treated using landfills or incineration methods cause serious environmental pollution, and hence, research on waste plastic recycling methods is of utmost importance.

Plastic has excellent durability and is lightweight, and the research on the methods of recycling it into building aggregates, based on its natural chemical stability and high impact resistance, is ongoing. In particular, when waste plastics are used as aggregates, substantial amounts of waste can be recycled, thereby solving the problem of aggregate shortages at construction sites and reducing the environmental impact caused by natural aggregate mining and treatment [2,3,4,5].

To date, various types of waste plastics such as polyethylene terephthalate (PET) [6,7,8,9], polyvinylchloride (PVC) [10], high-density polyethylene (HDPE) [11], expanded polystyrene (EPS) [12], polycarbonate (PC) [13], thermosetting plastic [14], and mixed plastic waste [15,16] have been used and studied as aggregates or fillers in the manufacture of cement mortar and concrete. ByFusion Global Inc. (Gardena, CA, USA) in the United States manufactures waste plastic bricks in an eco-friendly way by using hot water and high pressure without any adhesives or additives. They have reported that, compared to conventional concrete bricks, greenhouse gas generation is reduced by more than 95%, and since the waste plastics are immediately recycled and used, waste treatment is no longer needed, thus saving the costs for additional transportation and landfills. ‘Newatlas’, a Taiwanese venture company specializing in waste recycling, proposed new alternatives for building and interior materials using waste plastics. They reported that a recycled brick substitute called ‘Polli-Brick’ reduced the weight of a building by over 50%, and the translucent material saved electricity by increasing the natural lighting. ART (Advanced Recycling Technik GmbH, Wien, Austria) uses special extruders to produce architectural wood material substitutes with mixed waste plastics through a mixed recycling process. These wood substitutes have the advantage that they can be produced without the cleaning or removal of foreign substances by mixing all the melting materials without having to add other chemicals.

In addition, Jassim’s research team at the University of Basrah in Iraq used HDPE as fine aggregate in an eco-friendly waste treatment method to manufacture plastic cement within a range of 10–80% volume fraction [17]. They reported that the density decreased, the ductility increased, and the workability improved in the manufactured plastic cement. ‘Iridex Group Plastic’ in Romania developed a lightweight concrete based on waste plastics/rubber mixtures (polyurethane, waste tires, etc.). The developed concrete showed a density between 1050 and 1130 kg/m^3^ and a low thermal conductivity (0.20–0.33 W/mK) [18].

However, previous studies have shown that plastic, a hydrocarbon-based polymer compound, exhibits hydrophobicity properties, which slows the contact between water and cement powder and inhibits the hydration reactions when applied as aggregates [19,20]. Consequently, only weak bonds are formed between the plastic aggregate and the cement paste, and thus the bonding properties with the cement substrate are weak. In other words, the mechanical properties and the durability of concrete deteriorate, owing to the hydrophobicity of the plastic aggregate surface. Owing to these practical problems, the technology to apply waste plastic as an aggregate to concrete mixtures has not been actively practicalized or commercialized thus far, and active research and development are required to improve the interaction between the plastic aggregates and the cement paste.

In this paper, we conducted a study to modify the surface of aggregates for concrete based on waste composite films with the aim of improving the compatibility of plastic aggregates and cement substrates. To this end, we used waste composite films collected from actual household wastes to manufacture aggregates through extrusion molding. Then, we used ultraviolet–ozone treatment (UV-O_3_), a silane coupling agent, O_2_ atmospheric pressure plasma, and acrylic binder coating methods to modify the surface of the manufactured aggregates. The changes in the surface properties according to the treatment conditions were analyzed by measuring the contact angle. After identifying the rate of change in the contact angle over the time following the treatment, the conditions for optimizing the surface modification of the aggregate for concrete based on waste composite films were finally established.

## 2. Materials and Methods

### 2.1. Materials

The waste polymer films used in this experiment were obtained from Icheon, Gyeonggi-do, Korea on 21 June 2021, through Daeil Environment Co., Ltd. (Ichoen, Korea), a waste collection company. The blast slag fine powder (average particle diameter: 3.5 μm, density: 2.85 g/cm^3^) was provided from Hanaktec. Co., Ltd. (Ichoen, Korea) for use as an inorganic filler. (3-Aminopropyl)triethoxysilane (APS, 99%, Aldrich, Saint Louis, MO, USA) was used as a silane coupling agent for surface treatment, and ethyl acrylate binder (ORGAL UAD-3, UNISOL Chemicals Co., Ltd., Seongnam, Korea) was used as a water-soluble acrylic resin.

### 2.2. Fabrication of Recycled Plastic Film-Based Aggregates

**Figure 1:**
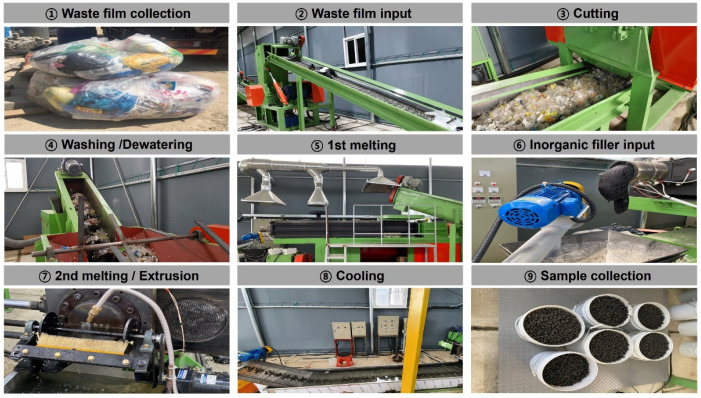
Production processes of recycled plastic film-based aggregates.

Figure 1 illustrates the entire process of manufacturing the recycled plastic film-based aggregate. The process is conducted in the following order: ‘waste plastic film collection’ → ‘cutting’ → ‘water washing’ → ‘dehydration’ → ‘primary melting’ → ‘input of inorganic filler’ → ‘secondary melting and extrusion’ → ‘cooling’ → ‘acquisition.’ Waste plastic films collected as household waste may cause problems in the regeneration process as they are mixed with other materials (such as scrap metal, paper, and glass) during discharge and storage; thus, they must be screened and removed using appropriate means. In this study, foreign substances present in the waste plastic film were removed through the primary manual screening process.

The waste film after the screening process was pulverized into an appropriate size for the qualitative homogenization of the final product and ease of process application. The cutting process was conducted through impact type, cutting type, pressing type, and shearing type grinders. In general, for thermoplastic polymers, cutting type grinders, and not shearing type grinders, are used owing to their high shear strain and ductility, and in special cases, cryogenic grinding is also used. In this study, the waste film was pulverized into a size of 100 mm or less using a cutting type of grinder.

The waste plastic film that was subjected to the cutting process was washed to remove pollutants through a water washing process. Wet washing was conducted based on friction and specific gravity differences. First, foreign substances such as soil and food were washed by spraying water onto the waste composite film before introducing it into the water tank. In the case of aluminum and scrap with a large specific gravity, flotation was performed based on the specific gravity difference in the water tank.

Moisture remaining in the wet-separated waste plastic film degrades the physical properties of the final product and causes a fatal problem in extrusion equipment during the process, and hence, a dehydration/drying process must be adopted. In this study, moisture was primarily removed through centrifugation, and as a secondary process, hot air drying was performed in a drum-type drier to remove the moisture from the wet-separated waste plastic film. The waste plastic film that was subjected to the drying process was processed into the final aggregate form through melting and extrusion molding. The waste plastic film, which was ground to a predetermined size after the washing/drying process, was placed in a hopper, which is a raw material inlet, through a screw conveyor device. The waste plastic film introduced into the melting machine was melted while passing through a single screw and was then transferred to the extruder. During this process, melting was conducted in the range of 330–370 °C, set during the thermal analysis of the waste plastic film, and the water vapor and organic gas discharged during the melting process were treated through a recovery device and then discharged through a cleaning tower.

The molten waste plastic film was introduced into an extruder with an inorganic filler. Inside the extruder, melting and roll mixing milling were conducted through the rotation of the heated twin screw, and finally, the uniformly mixed waste plastic film and the inorganic filler were extruded using a die. The waste plastic film escaped and expanded (i.e., die swell or extrudate swell) owing to the inherent viscoelastic properties of the polymer. In this study, the shape and size of the die were designed in consideration of these properties, and the final aggregate was manufactured in the size of the fine aggregate (5 mm or less) and thick aggregate (20 mm or less). Furthermore, because the size and shape of the aggregate are dependent on the type of die, the die shape can be designed to increase the surface roughness; for instance, circular, triangular, square, and cross shapes can be designed to increase the mechanical interactions between the cement paste and the recycled plastic film-based aggregate in the future.

The filament-shaped waste plastic film/inorganic filler molded body extruded through the die outlet was immediately placed in a cooling tank to fix the shape through a water-cooling process, and then moisture was removed through an air-cooling process. After the cooling process, the waste plastic film/inorganic filler molded body wound in the winding machine was cut to an appropriate size through a cutter and then collected in the form of aggregate.

### 2.3. Surface Modification of Recycled Plastic Film-Based Aggregates

**Figure 2:**
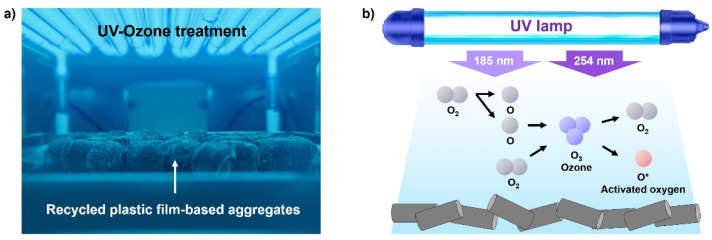
(**a**) Photograph and (**b**) schematic of the current work showing UV-O_3_ treatment of recycled plastic film-based aggregates. The 254 nm of UV light decomposes ozone (purple color) and produces activated oxygen (red color) with high energy.

The surface treatment technology using UV-O_3_ treatment is a simple method of increasing wettability and adhesion by introducing a polar functional group such as oxygen on the surface of a hydrophobic polymer [21,22]. Recently, this method has been widely used as a practical surface treatment method owing to its advantages, such as improved power of UV generating devices, miniaturization of devices, and environmental friendliness. Figure 2 depicts the principle of UV-O_3_ treatment. UV rays with a wavelength of 185 nm react with oxygen, generating O_3_. O_3_ is decomposed into oxygen and active oxygen (O*) by UV rays with a wavelength of 254 nm, and an oxide layer including an oxygen functional group is formed on the surface by active oxygen. In this work, we attempted to improve the hydrophilicity by forming an oxide layer on the hydrophobic surface of the recycled plastic film-based aggregate through UV irradiation. Under our experimental conditions, the UV-O_3_ treatment was performed with a UV intensity of 28 mW/cm^2^ (light source: grid type lamp, wavelength: 184.9 nm/253.7 nm) for 0–1200 s under nitrogen (N_2_) purging.

**Figure 3:**
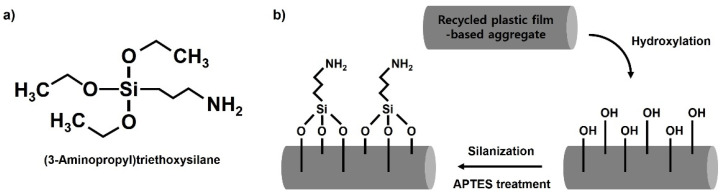
(**a**) Chemical structure of 3-aminopropyltriethoxysilane and (**b**) schematic illustration for the surface modification mechanism during silanization reaction.

A silane coupling agent is a material with two functional groups, one of which is compatible with a matrix, and the other is a functional group that can react with the surface of a given filler [23,24]. In general, silane coupling agents have a structure of X_3_SiR, where X denotes a chlorine atom or an alkoxy group and R denotes an organic functional group. X is hydrolyzed by moisture during the conversion to a silanol (–SiOH) group, which reacts with a hydroxyl group on the surface of the filler to form a siloxane (Si–O–Si) bond or hydrogen bond, thus modifying the surface. Meanwhile, R uses a coupling agent with various organic functional groups to form chemical bonds with the matrix or to have compatibility depending on the purpose. In this study, the surface of the waste composite film-based aggregate was hydrophilized using 3-aminopropyltriethoxysilane (APS), a silane coupling agent with a hydrophilic amine (–NH_2_) functional group as an organic functional group (Figure 3a). As depicted in Figure 3b, a hydrophilic amine functional group can be successfully introduced into the surface of the recycled plastic film-based aggregate through hydrolysis and condensation. In this study, the introduction of a functional group was conducted by changing the silane treatment time from 0 to 600 s.

**Figure 4:**
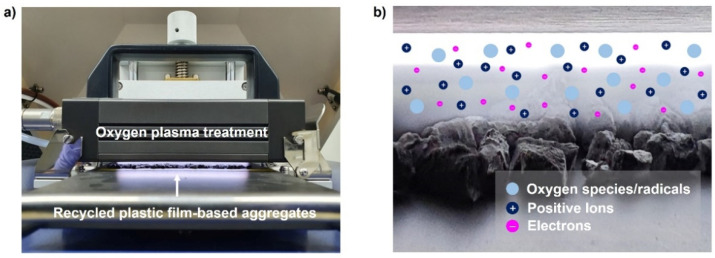
(**a**) Photograph and (**b**) schematics of the oxygen plasma treatment. Reactive oxygen species, radicals, positive ions, and electrons can be created by plasma discharge.

The atmospheric pressure plasma treatment is a method that can generate a functional group on a surface without changing the bulk characteristics of a material by generating uniform plasma under atmospheric pressure [25,26,27]. Such atmospheric pressure plasma has advantages in that it is simple, easy to use, and can overcome the weak economic feasibility and productivity of the existing vacuum plasma treatment method. When plasma generated after receiving energy comes in contact with oxygen molecules, oxygen ions (O^2+^) are generated, or molecules are separated into oxygen atom ions (2O^+^). In general, because the ionic state is highly unstable, it is combined with the surrounding electrons to become oxygen radical (O*) or is combined with other oxygen ions to form O_3_, and a hydroxyl group (·OH) is produced by the decomposition of water vapor in air. Oxygen atmospheric pressure plasma treatment is a representative surface modification method using oxygen chemically active species generated through multistage chemical reactions. Oxygen active species form various polar functional groups by combining with the material surface, and these polar groups result in high surface free energy and hydrophilicity characteristics. In this study, the oxygen atmospheric pressure plasma treatment used to modify the surface of the recycled plastic film-based aggregate is illustrated in Figure 4. The surface treatment was performed by injecting O_2_ and Ar gases at flow rates of 15 sccm and 4 sccm, respectively, with an RF power of 100 W under atmospheric pressure and by changing the processing time from 0 to 30 s.

**Figure 5:**
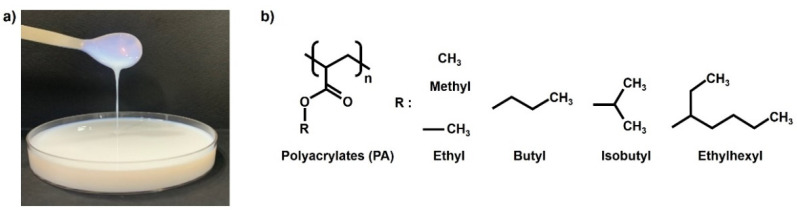
(**a**) Photograph of acrylic binder used in this work and (**b**) the chemical structures of representative polyacrylates.

An acrylic binder is formed by polymerizing various acrylate monomers with acrylic or methacrylic acid esters. The acrylic binder resin has been attracting increasing attention owing to its excellent durability and chemical resistance. In particular, water-soluble acrylic binders have significant application potential in the surface coating industry owing to their eco-friendliness and high economic feasibility. The chemical structural formulas of various water-soluble acrylic binders are depicted in Figure 5. Furthermore, acrylic binders are hydrophilic because they contain chemically high polar carboxyl groups. In this study, a hydrophilic acryl binder coating was conducted to contribute to the increase in adhesive force by increasing the polar surface energy of the surface of the recycled plastic film-based aggregate. The thickness of the coating layer was adjusted by changing the wetting time, and the experiments were performed while changing the wetting time from 0 to 300 s.

### 2.4. Equipments

All the equipment for the aggregate manufacturing (such as a grinder, washer, dehydrator, hot air dryer, primary/second extruder, water cooling/air cooling device, and cutter) were custom-made and used (Daesung Resin Machinery, Daegu, Korea). The waste composite film was extruded into an aggregate form while controlling the input speed, melting temperature, screw rotation speed, and reaction mixture ratio, using a twin-screw extruder. UV-O_3_ cleaner (AC-6, AhTech LTS Co., Ltd., Anyang-si, Korea) and O_2_ atmospheric pressure plasma (MyPL-100P(S), APP Co., Ltd., Hwaseong-si, Korea) were used for surface treatment.

### 2.5. Characterization

Changes in the surface properties of the waste composite film-based aggregates were analyzed using a contact angle measuring instrument (DSA25, KRÜSS Optronic GmbH, Hamburg, Germany). For the contact angle, the angle between 5 μL of deionized water droplets and the aggregate surface was measured under the conditions of 60% relative humidity and 20 °C.

## 3. Results and Discussion

### 3.1. Hydrophilicity Test According to Surface Modification Time

To investigate the hydrophilization effect through the various surface modification treatments, the change in the surface properties of the aggregates was indirectly determined through the change in the contact angle.

**Figure 6:**
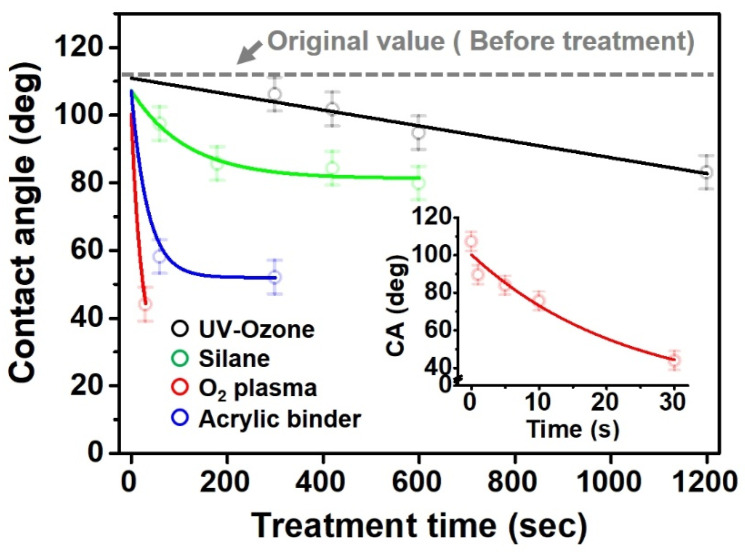
Contact angle change as a function of surface modification types and time for the recycled plastic film-based aggregates.

The contact surface treatment time varied in accordance with the surface modification method, and after surface modification, the change in the contact angle with the water droplets was measured five times and the average value was presented. The contact angle measurement results for each surface treatment time are summarized in Figure 6 and Table 1, respectively. The recycled plastic film-based aggregate exhibited a hydrophobic contact angle of approximately 108° when its surface was not modified. In general, as the contact angle of the material surface decreases, the surface energy increases, indicating an increase in wettability [28,29]. It was confirmed that in all the surface treatment methods conducted in this study, the contact angle decreased with an increase in the treatment time. This reduction in the contact angle may be attributed to the polar functional groups formed through the surface modification method. In these experiments, the final contact angles according to each maximum surface treatment time were measured to be 83.0°, 79.8°, 44.0°, and 52.0°, in the case of the UV-O_3_ treatment, the silane coupling agent, the oxygen atmospheric pressure plasma, and the acryl binder coating methods, respectively. In particular, the oxygen atmospheric pressure plasma treatment resulted in the lowest contact angle within an extremely short surface treatment time of 30 s. The comparison of the contact angles at the same surface treatment time revealed that oxygen atmospheric pressure plasma treatment was the most effective surface modification method for hydrophilicity.

### 3.2. Hydrophilicity Test over Time after Surface Modifications

The hydrophilicity of a polymer surface treated using various surface modification methods varies in accordance with several parameters such as the surrounding environment, temperature, and time [30,31]. This is believed to be due to the high chain mobility of the polymer alongside the possibility of the adsorption of heterogeneous atoms of activated species. In general, a polymer chain has greater mobility on the surface than on the inside, and hence, the polar functional groups formed by the surface modification are rearranged inside the materials, and the chains with low molecular weight are rearranged on the surfaces as the binding energy decreases [32].

**Figure 7:**
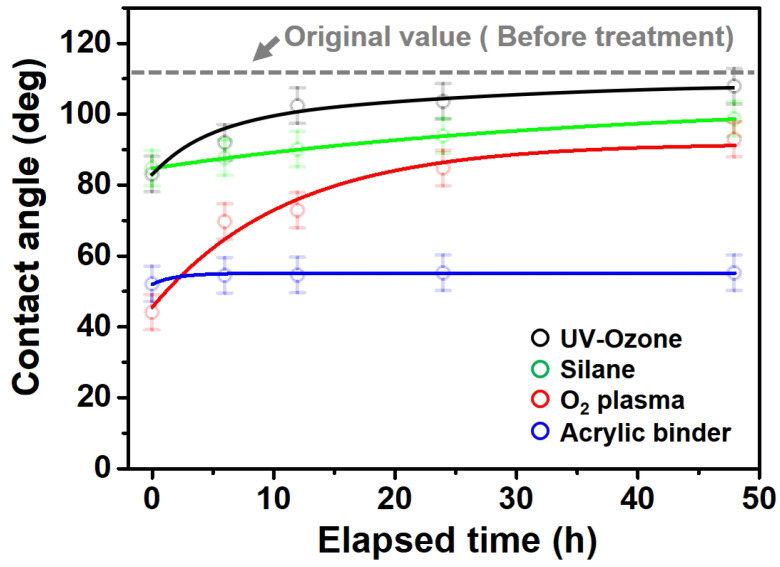
Contact angle change as a function of elapsed time for the recycled plastic film-based aggregates.

Figure 7 and Table 2 show the change in the contact angle over time when the waste composite film aggregate was treated using each surface modification method and stored in the atmosphere (0, 6, 12, 24, and 48 h). The experimental results indicated that the contact angle increased over time after surface modification in all samples, except for the sample subjected to the acrylic binder coating method. When the recycled plastic film-based aggregates with contact angles of 83.0°, 84.7°, and 44.0° were stored in the atmosphere after UV-O_3_ treatment, the silane coupling agent treatment, and the oxygen atmospheric pressure plasma treatment, the contact angles continued to increase over time and reached 107.8°, 98.6°, and 92.8°, respectively, after 48 h. The increasing rate rapidly increased during the initial few hours but decreased over time. Consequently, it was predicted that after a certain time, the contact angle in the atmosphere would be close to the contact angle of the aggregate in the initial untreated state. This increase in the contact angle over the lapse of storage time is determined to be the result of the rearrangement of surface polar functional groups and the interaction between heterogeneous atoms of adsorbed species in the atmosphere [33,34].

On the contrary, in the case of the recycled plastic film-based aggregates, which was modified using an acrylic binder, it was found that the surface contact angle was maintained almost the same. This is believed to be because, in the case of acrylic binders, the binder forms a physical bond in the form of a relatively thick film, unlike in the other surface modification methods. In general, a minimum work time of 5 days is required from the surface modification to the transportation and blending processes of the aggregate. If there is a change in the contact angle over time, the surface modification effect is significantly reduced, and thus, in future optimization experiments, surface modification will be performed using an acrylic binder with no change in the contact angle over time.

### 3.3. Optimization of Surface Modification of Recyled Plastic Film-Based Aggregates

**Figure 8:**
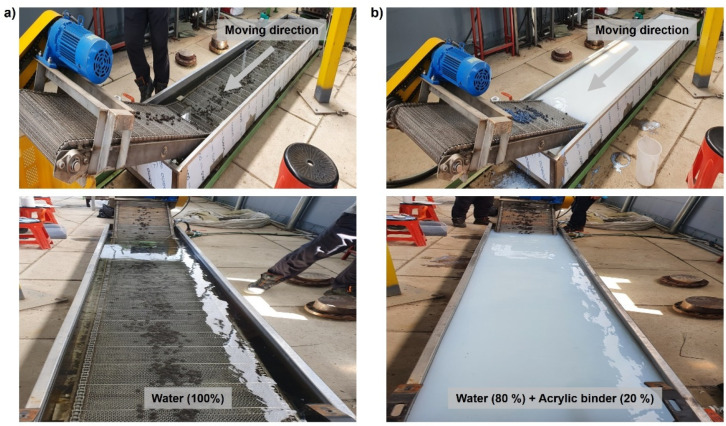
Photograph of the recycled plastic film-based aggregates production line (**a**) without and (**b**) with surface treatment.

Figure 8 illustrates an image in which an acrylic binder is applied to the actual surface modification process of the recycled plastic film-based aggregate. The recycled plastic film-based aggregate extruded through the die outlet was introduced into a cooling tank containing an acrylic binder to perform cooling and surface modification simultaneously. The wetting time varied from a minimum of 15 s to 180 s by adjusting the movement speed of the conveyor belt in the cooling tank; considering a smooth aggregate movement time, the wetting time was fixed to 100 s. Based on the results of the field application experiments, it was visually confirmed that the acrylic binder remained on the surface of the recycled plastic film-based aggregate.

**Figure 9:**
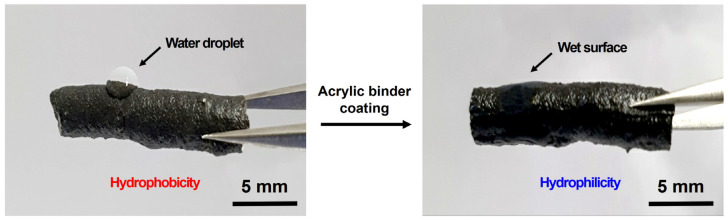
Photograph of the recycled plastic film-based aggregate before and after acrylic binder coating treatment. After treatment, an increase in hydrophilicity is observed.

Figure 9 depicts an image of the wettability of the recycled plastic film-based aggregate before and after treatment to determine the hydrophilization effect according to the surface treatment using an acrylic binder. When the surface modification was not performed, water molecules could not wet the surface of the aggregate and remained on the surface in the form of droplets. However, as the surface characteristics were modified to be hydrophilic through the surface treatment using the acryl binder, the overall wettability of the aggregate surface was improved after treatment.

## 4. Conclusions

In this study, changes in the surface properties of the aggregates were compared and analyzed using various surface modification methods with the aim of improving the compatibility between the recycled plastic film-based aggregates and the cement pastes. The surface of the aggregate produced through extrusion molding of the waste composite film was modified using UV-O_3_, a silane coupling agent, O_2_ atmospheric pressure plasma, and acrylic binder coating methods, and the change in the wettability according to the surface treatment method was analyzed by measuring the contact angle. It was confirmed that the treatment time was the most important factor in determining the degree of hydrophilicity. The contact angle of the recycled plastic film-based aggregates decreased with an increase in the treatment time in all the surface modification methods. Moreover, owing to the rearrangement of the surface polar functional groups and the interaction between heterogeneous atoms, the contact angle tended to increase again with the passage of storage time after the surface treatment. However, in the case of surface modification using an acrylic binder, the binder and the recycled plastic film-based aggregates formed a physical bond, and thus, there was no significant change in the contact angle even after a long time. Finally, the hydrophobic surface of the recycled plastic film-based aggregates could be successfully modified to be hydrophilic using an acrylic binder. Furthermore, based on the advantage of being able to conduct surface modification in a short processing time while cooling, the surface-modified aggregate could be mass-produced through a continuous process without damage to the bulk properties and the surface shape of the aggregate. Consequently, based on the results of this study, we expect that the applicability of waste plastic/waste vinyl materials will increase as aggregates for building construction, since not only are the disadvantages of conventional waste composite film-based aggregates overcome, but also a synergy effect can be achieved.

## Figures and Tables

**Table 1:** Contact angle measured on the recycled plastic film-based aggregates as a function of surface modification types and time.

Type of Surface Modification	Treatment Time				
UV-Ozone treatment	0 s	60 s	300 s	600 s	1200 s
	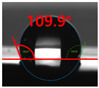	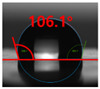	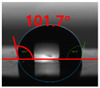	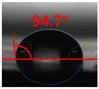	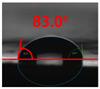
Silane coupling treatment	0 s	60 s	180 s	420 s	600 s
	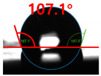	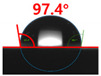	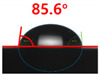	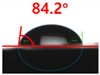	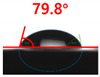
O_2_ plasma treatment	0 s	1 s	5 s	10 s	30 s
	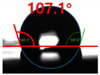	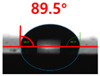	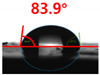	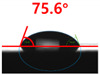	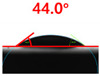
Acrylic binder coating	0 s	10 s	30 s	60 s	300 s
	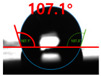	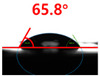	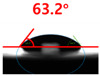	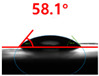	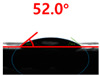

These contact angles were measured using a hanging 5 microliter drop of water.

**Table 2:** Effect of aging on contact angle for samples through various surface modification methods.

Type of Surface Modification	Aging Time				
	0 h	6 h	12 h	24 h	48 h
UV-Ozone treatment	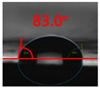	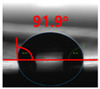	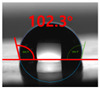	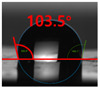	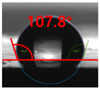
Silane coupling treatment	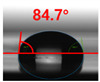	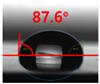	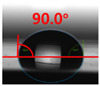	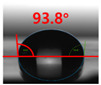	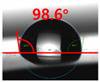
O_2_ plasma treatment	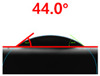	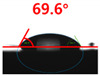	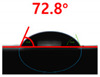	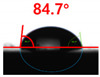	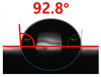
Acrylic binder coating	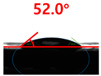	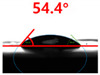	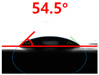	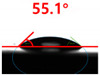	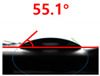

These contact angles were measured using a hanging 5 microliter drop of water.

## Data Availability

Data sharing is not applicable to this article.
